# Cancer-Related Stigma in Malawi: Narratives of Cancer Survivors

**DOI:** 10.1200/GO.22.00307

**Published:** 2023-02-16

**Authors:** Melissa H. Watt, Gita Suneja, Chifundo Zimba, Katherine D. Westmoreland, Agatha Bula, Lux Cutler, Abhilasha Khatri, Matthew S. Painschab, Stephen Kimani

**Affiliations:** ^1^ Department of Population Health Sciences, University of Utah, Salt Lake City, UT; ^2^ Department of Radiation Oncology, University of Utah, Salt Lake City, UT; ^3^ UNC Project Malawi, Lilongwe, Malawi; ^4^ Department of Pediatric Hematology-Oncology, University of North Carolina at Chapel Hill, Chapel Hill, NC; ^5^ Honors College, University of Utah, Salt Lake City, UT; ^6^ School of Biological Sciences and Honors College, University of Utah, Salt Lake City, UT; ^7^ Department of Hematology, University of North Carolina at Chapel Hill, Chapel Hill, NC; ^8^ Department of Medicine, Division of Medical Oncology, University of Utah, Salt Lake City, UT

## Abstract

Stigma is an impediment across the cancer care continuum, leading to delayed presentation to care, elevated morbidity and mortality, and reduced quality of life. The goal of this study was to qualitatively examine the drivers, manifestations, and impacts of cancer-related stigma among individuals who received cancer treatment in Malawi, and to identify opportunities to address stigma.

**METHODS:**

Individuals who had completed treatment for lymphoma (n = 20) or breast cancer (n = 9) were recruited from observational cancer cohorts in Lilongwe, Malawi. Interviews explored the individual's cancer journey, from first symptoms through diagnosis, treatment, and recovery. Interviews were audio-recorded and translated from Chichewa to English. Data were coded for content related to stigma, and thematically analyzed to describe the drivers, manifestations, and impacts of stigma along the cancer journey.

**RESULTS:**

Drivers of cancer stigma included beliefs of cancer origin (cancer as infectious; cancer as a marker of HIV; cancer due to bewitchment), perceived changes in the individual with cancer (loss of social/economic role; physical changes), and expectations about the individual's future (cancer as death sentence). Cancer stigma manifested through gossip, isolation, and courtesy stigma toward family members. The impacts of cancer stigma included mental health distress, impediments to care engagement, lack of cancer disclosure, and self-isolation. Participants suggested the following programmatic needs: community education about cancer; counseling in health facilities; and peer support from cancer survivors.

**CONCLUSION:**

The results highlight multifactorial drivers, manifestations, and impacts of cancer-related stigma in Malawi, which may affect success of cancer screening and treatment programs. There is a clear need for multilevel interventions to improve community attitudes toward people with cancer, and to support individuals along the continuum of cancer care.

## INTRODUCTION

Stigma toward people with cancer creates barriers across the cancer care continuum, and leads to elevated morbidity and mortality.^1-3^ Link and Phelan^4^ define stigma as a cognitive separation of us and them groups, which leads to labeling, stereotyping, and separation. Common drivers of stigma across disease conditions and populations include negative attitudes about the patient population, fear of infection or contamination, association of the condition with unacceptable behaviors, and institutional norms and culture.^5^

**CONTEXT**

**Key Objective**
How do people with cancer in a low-income country setting experience stigma, and how does this affect their well-being? What can be done to address cancer-related stigma?
**Knowledge Generated**
Stigma was a universal experience for participants in this setting, leading to social, psychological, and clinical impacts. Participants highlighted the need for community education and peer support.
**Relevance**
Understanding cancer stigma can help to inform the design of comprehensive programs to support individuals along the cancer continuum, and improve cancer outcomes.

An emerging body of research from African countries highlights the presence and impact of cancer-related stigma. For cancers where routine screening exists (most notably, cervical cancer), there is evidence that people avoid screening because of stigmatization of individuals with cancer.^6-8^ For individuals diagnosed with cancer after symptom presentation, diagnosis narratives suggest that stigma plays a role in delayed presentation to care.^3^ Across multiple studies, women eventually diagnosed with breast cancer said that they delayed seeking a diagnosis because of fear of social exclusion.^9-11^ Once diagnosed, both internalized stigma (feelings of shame and lack of self-worth) and anticipated stigma (fear of discrimination of exclusion) complicate linkage to treatment and completion of treatment,^12,13^ even when other barriers of access are reduced. Reductions in cancer-related stigma have been shown to lead to improved cancer-specific psychological distress^14^ and increased willingness to undergo cancer screening.^15^

As elements of cancer care are becoming more common in African countries, and cancer is seen as a priority for research and intervention,^16^ it is essential to understand and address cancer stigma. The goal of this study was to examine the drivers, manifestations, and impacts of cancer-related stigma among individuals who received treatment for breast cancer or lymphoma in Malawi, and to identify opportunities to address cancer-related stigma.

## METHODS

This descriptive qualitative study involved in-depth interviews with survivors of lymphoma (n = 20) or nonmetastatic breast cancer (n = 9) in Malawi, both well-established cohorts in Malawi. The protocol was approved by the Malawi National Human Subject Research Council, and the ethical review boards of the University of North Carolina at Chapel Hill, and the University of Utah.

### Setting

The study was conducted at Kamuzu Central Hospital (KCH), a referral hospital located in Lilongwe, the capital of Malawi. KCH provides cancer diagnostic and treatment services to a catchment area of approximately nine million people. It is one of two public referral hospitals providing comprehensive cancer treatment in Malawi.

### Sample and Recruitment

Individuals were eligible to participate if they were at least age 18 years; had a history of biopsy-proven breast cancer or lymphoma; had completed curative-intent treatment and were currently in remission; were at least one year from cancer-directed treatment (ongoing adjuvant endocrine treatment was allowed for breast cancer survivors); and spoke fluent English or Chichewa. Participants were recruited from previously described existing observational cohorts of patients with breast cancer^17^ and lymphoma^18^ at KCH. Participants had received treatment according to previously described KCH standards of care for breast cancer^19^ and lymphoma,^18,20^ which are modeled after the National Comprehensive Cancer Network Harmonized Guidelines for Sub‐Saharan Africa.^21^ Remission was defined as the disappearance of all evident disease assessed by physical examination, chest radiography, abdominal sonography, and rarely CT scan. A research assistant contacted eligible participants via phone or approached them after their regularly scheduled clinic visit. Participants were informed about the study and provided written informed consent. Participants were purposively selected from existing observational cohorts of patients with breast cancer and lymphoma at KCH, to provide a balance of age and sex, and to include individuals who will be good informants (ie, articulate, reflective, and willing to share their own experiences).

### Data Collection

A semistructured interview guide was developed to elicit personal narratives of experiences along the cancer continuum, with a focus on the impact of cancer diagnosis and treatment on the individual's quality of life. The guide did not have specific questions or probes about stigma, but the open-ended nature of the interview included ample opportunity for discussion of stigma to arise organically.

Interviews were conducted by a Malawian researcher, who was fluent in both English and Chichewa and had significant training and experience in qualitative methods. The interview was scheduled for a time and place that was mutually convenient and maximized privacy. Because of the COVID-19 pandemic, some interviews (11/29) were conducted via telephone. The interviews lasted on average 55 minutes, with no significant difference between interviews conducted in person and interviews conducted via telephone (*P* = .1176). All interviews were audio-recorded, simultaneously transcribed, and translated from Chichewa into English.

### Analysis

Data were analyzed using applied thematic analysis^22^ with qualitative memo writing.^23^ Applied thematic analysis is a rigorous set of inductive procedures designed to identify and examine themes from textual data in a way that is transparent, reproducible, and credible. To extract meaning and make meaningful connections both within and across transcripts, detailed memos were written for each interview. The memo template was organized around the cancer care continuum (cancer diagnosis, initiation of treatment, and retention in treatment). For each section, themes were summarized related to barriers and facilitators, retaining representative quotations to support the inductive themes. The memos were written by two members of the research team (L.C. and A.K.), and each transcript/memo pair was discussed and revised with two other analysts (M.H.W. and S.K.) to confirm the completeness and rigor of the memos. On average, each full transcript represented 16 pages of single-spaced text and was condensed to six pages of text in the memo writing process.

Through the memo writing process, emerging themes were identified related to cancer stigma, which informed the development of a structured codebook. The codebook included four parent codes to represent the domains of interest (drivers of stigma; manifestations of stigma; impact of stigma; and opportunities to address stigma), and child codes under each domain captured emerging themes. The 29 document memos were uploaded to NVivo software and coded by the first author (M.H.W.); additional child codes were added as needed. Code-level queries were run, and the content was discussed to reach study team consensus on the final themes.

## RESULTS

### Description of the Sample

The sample was predominantly female (59%), had a median age of 45 years (IQR, 38-49), and at time of the interview were 3 years (IQR, 3-4) from their initial diagnosis (Table 1). All participants were diagnosed with cancer after the emergence of symptoms; none reported participation in routine screening for cancer.

**TABLE 1:**
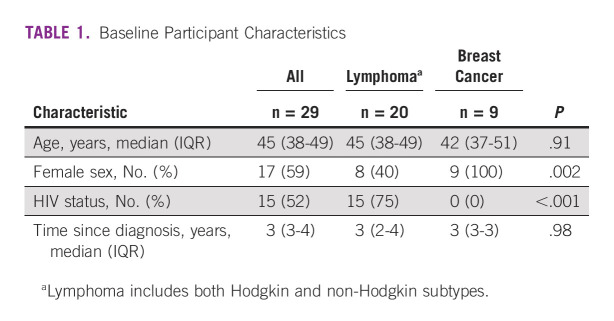
Baseline Participant Characteristics

**FIG 1:**
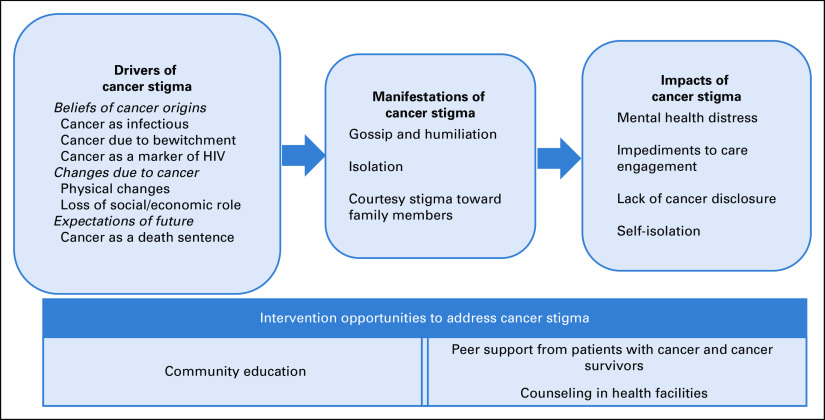
Summary of emerging themes.

Figure 1 summarizes the themes in the domains of drivers, manifestations, and impacts of cancer stigma, and Table 2 provides representative quotes. Below the emerging themes across each of the domains are described.

**TABLE 2:**
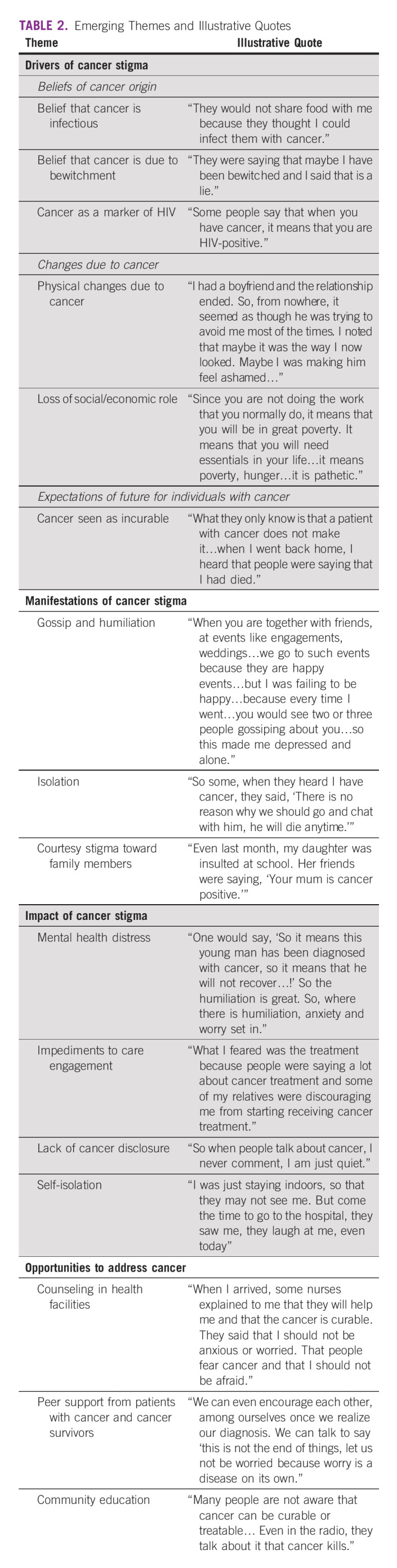
Emerging Themes and Illustrative Quotes

### Drivers of Cancer Stigma

Patient narratives suggested that cancer stigma was driven by (1) community beliefs about the origin of cancer, (2) the changes experienced by people with cancer, and (3) expectations of the future for people with cancer.

Beliefs about the origins of cancer included that cancer was infectious, caused by bewitchment, and a marker of HIV. These beliefs led to a process of othering and isolating people with cancer. Multiple participants explained how people in their community avoided close physical contact with them after their cancer diagnosis, because of fear of acquisition.

“The people in my community and my family found it difficult when they heard I had cancer. At times, they would fail to come to my house to visit me because they thought that ‘when we visit her, she will transmit the disease to us.’” (Female, 46 years old, breast cancer survivor)

Community beliefs that cancer was due to bewitchment led to community members speculating about the individual's personal character, and suggesting faith-based healing practices in lieu of medical treatment. A lack of community-level knowledge about cancer, and a belief that cancer was only experienced by individuals with HIV, meant that individuals with cancer also bore the brunt of HIV stigmatizing attitudes and behaviors.

“Some were laughing at me, when they saw me losing weight, changing my body color, my complexion had changed, so some people were laughing at me they were saying that, ‘This one is HIV-positive,’ but I am HIV-negative. So, all these things were giving me stress.” (Female, 41 years old, breast cancer survivor)

Stigma was further driven by the changes experienced by people with cancer. Physical changes included weight loss, hair loss, and obvious presence of tumors. Social and economic changes included less mobility, less independence, and a curtailed ability to earn money and support oneself and one's family. These changes due to cancer brought the participant unwanted and negative attention, and made them feel as if they were no longer integral members of their community.

“Since I was not doing anything, I was depending on people to assist me. It was difficult for me to tell them everything I needed; I still thought about things I would normally do for myself if it were not for my illness.” (Male, 47 years old, lymphoma survivor)

Finally, stigma was driven by community expectations that cancer is incurable and individuals with cancer will die of their disease. These community beliefs led participants to feel disregarded and dismissed, as they felt they were seen as having no future. The following participant described how his experience of being diagnosed with cancer was harder than his experience being diagnosed with HIV, because there was no public knowledge about cancer treatment.

“When I was diagnosed with HIV, I also had challenges with it, but in the end, I accepted it and I started taking ARVs [antiretrovial medication]. But when I was diagnosed with cancer, a condition which I had never heard anyone surviving, I was shattered… It was believed that once someone was diagnosed with cancer, it was a death sentence because there was not any treatment.” (Male, 45 years old, lymphoma survivor)

### Manifestations of Cancer Stigma

Participants identified three primary manifestations of stigma: (1) gossip and humiliation, (2) isolation, and (3) stigma toward family members. Gossip was the most common form of stigma discussed. Most gossip pertained to the individual's physical changes and therapeutic nihilism.

“So when they are discussing in these groups, they say, ‘That is the guy who was diagnosed with cancer.’ They also heard that cancer is not curable. So one would say, ‘So it means this young man has been diagnosed with cancer, so it means that he will not recover…!’ The humiliation is great.” (Male, 38 years old, lymphoma survivor)

Participants also noted that people in their community avoided them or stopped spending time with them. They related this to community beliefs that cancer was infectious and that death was inevitable, as this participant related, “So some when they heard I have cancer, they said, ‘There is no reason why we should go and chat with him, he will die anytime.’” (Male, 47, lymphoma survivor) A few participants also noted that stigma against them was extended to their family members, who were gossiped about or avoided due to living with someone with cancer.

### Impact of Cancer Stigma

In participant narratives, cancer-related stigma affected individuals in four key ways: (1) mental health distress, (2) impediments to care engagement, (3) lack of cancer disclosure, and (4) self-isolation. Almost all participants noted that the process of othering they experienced due to cancer affected them emotionally. Participants described feelings of sadness, anxiety, loneliness, and hopelessness.

“When you are together with friends at events like engagements, weddings…we go to such events because they are happy events…but I was failing to be happy, because every time I went, you would see two or three people gossiping about you. So this made me depressed and alone.” (Female, 38 years old, breast cancer survivor)

Several participants noted that the stigma they experienced created barriers for accepting their cancer diagnosis, and subsequently engaging in cancer treatment. In some cases, this had to do with feeling hopeless and unsupported; in other cases, it involved active discouragement from community members.

“What I feared was the treatment, because people were saying a lot about cancer treatment, and some of my relatives were discouraging me from starting cancer treatment.” (Female, 46 years old, lymphoma survivor)

The negative attitudes that existed in the community toward people with cancer led individuals to hide their cancer diagnosis, and anticipated stigma led individuals to self-isolate to avoid negative experiences. The following participant described how the stigma he experienced was almost as bad as the cancer itself, leading him to isolate from his community to keep his cancer secret.

“My life changed in that I started struggling with other things and not the sickness. I had been mocked, stigmatized, and gossiped about. A lot happened. I accepted that I was like that. I then told my guardians at the time not to tell anyone what I was suffering from. I feared that I would die from depression and not the cancer itself. I asked them to keep things confidential. I would go to the (cancer) clinic alone on Monday and return home on Tuesday after chemo. I felt that I was handling this alone and felt that there was no need to tell other people.” (Male, 45 years old, lymphoma survivor)

### Opportunities to Prevent and Mitigate Cancer Stigma

When participants were asked how people with cancer could be supported, three themes emerged related to opportunities to prevent and mitigate cancer stigma. At the community level, participants noted the need to provide education about the clinical origins of cancer and therapies available to treat and cure cancer. For patients receiving cancer treatment, participants discussed the importance of counseling, and connecting patients to cancer survivors, who can bear witness to the cancer experience and provide motivation and support. Several participants noted that, as survivors, they would be willing to be part of a support system for newly diagnosed patients undergoing cancer treatment.

“As a cancer survivor, I would tell the cancer patients or those on treatment that they should not worry and they will be fine. And I (will share that I) have experience, ‘Look at how I am,’ things like those. And personally, I can be happy if you can call me at any time so that I should come and sensitize these people so that they should be encouraged… I feel that if someone is advising you, you feel as though he/she is insulting you, because maybe you feel they have never suffered [from that illness]. But if they met with a survivor and tell them that they have been through that, and show them the evidence like the hair and things like that, I feel they would be encouraged.” (Female, 41 years old, breast cancer survivor)

## DISCUSSION

In many low- and middle-income countries (LMICs), cancer is only recently emerging as a diagnosable, treatable, and survivable condition. In this study in Malawi, cancer survivors recounted experiences of cancer-related stigma throughout their cancer journeys. Cancer stigma was driven by misbeliefs about the origin of cancer, changes in the lives of people with cancer, and the association of cancer with certain death. Addressing cancer-related stigma is crucial because, as the data suggest, stigma affects patients' well-being, their engagement in cancer care, and their ability to harness social support.

The stigmatizing attitudes and behaviors experienced by people with cancer in Malawi harkens to the early days of the HIV epidemic, where an HIV diagnosis was associated with near-certain mortality, and communities were fearful about HIV transmission via casual contact.^24^ Participants in the study noted that the physical symptoms they experienced as a result of cancer led people to assume they were living with HIV, likely driven by the community experience of HIV-related illness before universal access to antiretroviral therapy in Malawi. That said, HIV stigma has improved considerably in African countries, with the advent of treatment and survivorship.^25^ As an indicator of this, one participant in the study who was dually diagnosed with HIV and cancer shared that his diagnosis with HIV was much easier to accept because it was well known that treatments for HIV were freely available. This was in contrast to cancer, where the availability of treatment in low-resource settings is new and unknown to communities. The experience of HIV stigma offers hope that cancer stigma will improve as more people are diagnosed and survive, and as medical care becomes more available.

The well-being of people with cancer is assessed not only in their survival, but also in their quality of life. In the United States, individuals with cancer often experience functional impairment, psychosocial distress, unemployment, financial toxicity, and overall poor quality of life.^26-28^ Social support is important to buffer these impacts, and in turn to support long-term commitment to and engagement in cancer care.^29^ As noted in the data, cancer-related stigma prevented individuals from mobilizing social support, leading instead to patients keeping their cancer secret and isolating themselves from their families and communities. Addressing cancer-related stigma will directly improve the quality of life of patients with cancer, and support their long-term care engagement.

To address the origins and impacts of cancer-related stigma, participants noted the value of community education, clinic-based counseling, and peer support programs. Community education and sensitization campaigns have had major impacts in high-income countries, including efforts such as the Pink Ribbon campaign for breast cancer.^30^ In low-income countries, community campaigns have effectively improved stigmatizing attitudes related to HIV^31^ and mental health ^32^; lessons from these community campaigns can be harnessed to address stigma related to cancer. In addition, clinic-based counseling strategies are essential at the time of testing and subsequent diagnosis to help an individual accept their diagnosis and cope with both internalized and anticipated stigma. Multiple participants suggested the need for peer support programs that involve cancer survivors as mentors and navigators. They note the value of lived experiences with cancer, and the importance of role modeling survivorship to provide a sense of hope and build resilience in the face of community stigma. In high-income countries, peer navigation has proven to reduce distress from cancer symptoms, enhance emotional well-being and satisfaction, and improve care coordination.^33-37^ Although peer navigation is well studied in Africa, these models have thus far not been applied to supporting individuals undergoing cancer treatment.^38^

This study brings to light the voices and experiences of individuals with cancer in Malawi. Nevertheless, the findings must be interpreted in light of the limitations. First, individuals who participated in this study had completed cancer treatment, and therefore, found ways to overcome stigmatizing attitudes and experiences. Individuals who failed to initiate treatment, or who subsequently dropped out of care, may have had very different experiences with cancer-related stigma that were not represented in this study. Second, the interview guide was not designed to ask or probe around stigmatizing experiences. The fact that stigma emerged so forcefully speaks to the impact of stigma in this setting; however, it is possible that more directed questions about stigma would have elicited more information and allowed us to develop a more accurate taxonomy of cancer-related stigma in this setting. Third, the audio-recorded interviews were transcribed from Chichewa to English but were not back-translated, which may have resulted in a loss of nuanced meaning of the construct of cancer-related stigma. Fourth, the study included participants from existing observational cohort studies with only two tumor types who received complete clinicopathologic and therapeutic care at a tertiary referral hospital. This limits the generalizability of our study findings. However, given that breast cancer and lymphoma share important attributes as well as key differences pertaining to treatment, their association with HIV, and survival outcomes, this study design made it possible to explore how cancer-related stigma had commonalities across cancer experiences. Finally, qualitative interviews are always subject to social desirability bias. The fact that the study was conducted in collaboration with the health care facility where individuals received cancer care may have resulted in participants moderating their experiences, and in particular, withholding experiences of stigma within the health care setting.

In conclusion, addressing cancer-related stigma is a priority to support the success of cancer prevention and treatment programs in LMICs. Multilevel interventions to address the drivers, manifestations, and impacts of cancer-related stigma are urgently needed. Community education campaigns, facility-based counseling, and peer navigation models hold promise to address cancer stigma. The perspectives and experiences of cancer survivors must be used to implement and disseminate strategies to eliminate cancer stigma.
